# Editorial: Canine lymphoma pathogenesis, diagnosis, prognosis and treatment: current and future perspectives

**DOI:** 10.3389/fvets.2024.1442964

**Published:** 2024-07-16

**Authors:** Vittoria Castiglioni, Monica Sforna, Cynthia de Vries

**Affiliations:** ^1^IDEXX Laboratories Italia Srl, Milano, Italy; ^2^Dipartimento di Medicina Veterinaria, Patologia Generale e Anatomia Patologica Veterinaria, University of Perugia, Perugia, Italy; ^3^Laboklin GmbH & Co. KG, Kissingen, Germany

**Keywords:** canine lymphoma, pathogenesis, diagnostic tools, therapy, artificial intelligence

Tumors of the hemolymphatic system are divided into lymphoid (lymphoma and leukemia) and myeloid leukemias, with lymphomas arising in lymphoid tissues outside the bone marrow and leukemias arising in the bone marrow or spleen (1). Canine lymphoma itself encompasses a heterogeneous group of malignancies, with different underlying molecular landscapes, clinical presentations, histologic distribution, morphology of cells, immunophenotype, prognosis, therapies, and outcomes. The Kiel and WHO/REAL classifications use multiple parameters to subdivide lymphomas into many categories and there is compelling evidence of the effectiveness of molecular techniques, which are gaining a primary role and complementing these morphologic classifications. There have been significant changes in the diagnosis, staging, classification, and treatment of canine lymphoma over the past few years. These improvements are due in large part to advances in basic understanding of the disease and novel therapies, particularly those used to treat the most aggressive subtypes.

In light of these considerations, lymphoma might be regarded as a fertile starting point for different experts, including internal medicine specialists, clinical and anatomic pathologists, oncologists, diagnostic imaging experts, and surgeons, to profitably discuss, confront, and provide their insights and perspectives. Furthermore, the increasing understanding of the biology of lymphomas developing in nonhuman species has created enthusiasm around the use of animal models that might recapitulate the human disease in more biologically pertinent ways. The development of lymphoma in companion pets, namely dogs, for example, displays many similarities with the human disease, so pets could serve as a meaningful model to improve translational drug development while concomitantly creating new treatment opportunities (2).

We include in the following paragraphs a brief summary of the main findings reported by different authors in their nine manuscripts that form this Research Topic.

Unfortunately, many types of cancer are detected at an advanced stage, when treatment options are limited and prognosis is poor. Early detection is an essential key point in the diagnosis of cancer, substantially increasing survival rates. Addressing this compelling need for cost-effective, simple, rapid, and non-invasive tests and novel diagnostic tools to be validated, up to five reviews of this Research Topic focus on early diagnostic biomarkers for canine lymphomas.

Sharif et al. validated an ELISA test intendent to measure a canine serum, Thymidine Kinase 1 (TK1), a protein involved in the pathogenesis of lymphoma, and compare its levels among dogs harboring lymphoma and healthy dogs. A statistically significant difference was found between these two groups, placing the canine TK1 ELISA test as an efficient screening tool. On this track, Olayinka et al. identified novel RNA biomarkers as an attempt to have repeatable tools to distinguish early-stage cutaneous T-cell lymphoma (CTCL) from interface dermatitis, with interesting parallelisms with the human counterpart. These are promising instruments to develop targeted therapies and treatment responses for both veterinary and human cutaneous T cell lymphomas.

Hammer et al. focused on MicroRNAs (miRNA, small non-coding RNAs), physiologically occurring small non-coding RNA molecules amounting to ~18–25 nucleotides that are not able to code for proteins but are involved in regulating gene expressions in the post-transcription process. Dysregulated miRNAs in canine lymphomas were evaluated, exploiting their diagnostic utility and highlighting different expression profiles not only between healthy vs. lymphoma-bearing patients but also among different canine lymphoma subtypes.

Next-generation sequencing (NGS) technology was explored by Fibi-Smetana et al. as a promising molecular tool for characterizing and stratifying canine lymphoma patients. NGS has numerous applications, ranging from whole-genome (re)sequencing to targeted sequencing for variant identification or confirmation. In contrast to whole-genome sequencing, targeted NGS focuses on a specific set of genomic loci that are likely to be involved in the phenotype of interest, delivering higher coverage levels at a more affordable cost, and making it amenable to samples containing small DNA amounts. In detail, the authors developed a targeted sequencing panel for canine lymphoma (TiHoCL), comprising ~100 canine loci, with encouraging results.

Moving away from molecular biology, Robertson et al. validated a screening test to be performed on canine urine samples, which was able to provide a fingerprint of urine of dogs harboring malignancies, one of which was lymphoma, different from that observed in healthy individuals. This test, known as Raman spectroscopy, has a 94% sensitivity and 90.5% specificity in evaluating metabolomes in urine in a simple, non-invasive, and rapid way.

An *in vitro* assay was presented by Hernández-Suárez et al., detailing changes in components of DNA Damage Response (DDR) in lymphoma cell lines. DDR is one of the pathways whose dysfunction can lead to cancer and resistance to genotoxic stress but might also present an opportunity to be used as a target for anticancer therapies.

An overview on new therapeutical protocols is offered by Lai et al., evaluating the efficacy of two treatments (L-LOP and L-LOPP) for the treatment of canine gastrointestinal (GI) and hepatosplenic (HS) high-grade lymphomas, traditionally associated with a very poor response to chemotherapy and, consequently, poor prognosis. These new suggested protocols turned out to be well tolerated with mild and transient adverse events and longer median survival time and progression-free survival. Moving to B-cell lymphomas, André et al. investigated a promising targeted drug delivery system through liposomes in the treatment of canine B-cell lymphomas, an immunophenotype that accounts for ~80% of canine lymphomas (3, 4).

Finally, Hubbard-Perez et al. looked to the (close) future, with the application of artificial intelligence (AI) to digital pathology. Deep learning (DL) through convolutional neural networks (CNNs) was applied to distinguish between normal, hyperplastic, and lymphoma-bearing lymph nodes and to discriminate among three common WHO subtypes of canine lymphoma, providing promising results.

Collectively, this Research Topic highlighted current research activities and trends in canine lymphoma, providing a state of art of the tools currently available in diagnosis, prognosis, and treatment and giving insights into the new advances for canine lymphoma in comparative oncology.

## Author contributions

VC: Conceptualization, Writing – original draft. MS: Writing – review & editing. CV: Writing – review & editing.
